# CD226 and TIGIT Cooperate in the Differentiation and Maturation of Human Tfh Cells

**DOI:** 10.3389/fimmu.2022.840457

**Published:** 2022-02-22

**Authors:** Motoko Yasutomi, Allison F. Christiaansen, Naoko Imai, Natalia Martin-Orozco, Christian V. Forst, Gang Chen, Hideki Ueno

**Affiliations:** ^1^ Department of Microbiology, Icahn School of Medicine at Mount Sinai, New York, NY, United States; ^2^ EMD Serono Research and Development Institute Inc. (The Healthcare Business of Merck KGaA, Darmstadt, Germany), Billerica, MA, United States; ^3^ Department of Genetics and Genomic Sciences, Department of Microbiology, The Icahn Institute for Data Science and Genomic Technology, New York, NY, United States; ^4^ Department of Immunology, Graduate School of Medicine, Kyoto University, Kyoto, Japan; ^5^ ASHBi Institute for the Advanced Study of Human Biology, Kyoto University, Kyoto, Japan

**Keywords:** TIGIT, CD226 (DNAM-1), T follicular helper (Tfh) cell, human, CD155

## Abstract

Costimulation pathways play an essential role in T cell activation, differentiation, and regulation. CD155 expressed on antigen-presenting cells (APCs) interacts with TIGIT, an inhibitory costimulatory molecule, and CD226, an activating costimulatory molecule, on T cells. TIGIT and CD226 are expressed at varying levels depending on the T cell subset and activation state. T follicular helper cells in germinal centers (GC-Tfh) in human tonsils express high TIGIT and low CD226. However, the biological role of the CD155/TIGIT/CD226 axis in human Tfh cell biology has not been elucidated. To address this, we analyzed tonsillar CD4^+^ T cell subsets cultured with artificial APCs constitutively expressing CD155. Here we show that CD226 signals promote the early phase of Tfh cell differentiation in humans. CD155 signals promoted the proliferation of naïve CD4^+^ T cells and Tfh precursors (pre-Tfh) isolated from human tonsils and upregulated multiple Tfh molecules and decreased IL-2, a cytokine detrimental for Tfh cell differentiation. Blocking CD226 potently inhibited their proliferation and expression of Tfh markers. By contrast, while CD155 signals promoted the proliferation of tonsillar GC-Tfh cells, their proliferation required only weak CD226 signals. Furthermore, attenuating CD226 signals rather increased the expression of CXCR5, ICOS, and IL-21 by CD155-stimulated GC-Tfh cells. Thus, the importance of CD226 signals changes according to the differentiation stage of human Tfh cells and wanes in mature GC-Tfh cells. High TIGIT expression on GC-Tfh may play a role in attenuating the detrimental CD226 signals post GC-Tfh cell maturation.

## Introduction

T cell activation requires a balance of stimulation and inhibition. Positive signals are required for T cells to fight against pathogens, while negative signals are required to prevent damage due to excessive inflammation. Costimulatory and coinhibitory signals serve as key regulators of this equilibrium. The most well-studied pair are CD28 and CTLA-4. Both molecules receive signals from CD80 and CD86 on antigen-presenting cells (APCs) to either induce T cell activation in the case of CD28 or inhibit T cell and APC activation in the case of CTLA-4 (1). CD155 and CD112 also play dual roles in regulating T cell activation by inducing T cell activation through the costimulatory molecule CD226 (DNAM-1) and inhibiting activation through TIGIT (T cell immunoglobulin and immunoreceptor tyrosine-based inhibitory motif ITIM domain). TIGIT deficiency or blockade results in enhanced T cell activation and exacerbated disease in experimental autoimmune encephalitis (EAE) (2–4), whereas it allows for enhanced tumor clearance in murine myeloma models and prevented metastasis (5, 6). In humans, increased TIGIT expression in cancer is associated with worse disease outcomes and TIGIT blockade and siRNA knockdown in T cells restores their effector functions (7, 8). In contrast, CD226 deficiency reduces disease susceptibility in EAE and inhibits tumor clearance (9–11). CD226 polymorphisms are also associated with a number of autoimmune diseases, including type 1 diabetes, rheumatoid arthritis, systemic lupus erythematosus, and Sjogren’s syndrome (12–15). Thus, TIGIT and CD226 play opposing roles in disease development.

TIGIT and CD226 compete for binding to their ligand CD155, however, TIGIT has a higher binding priority than CD226 (4). CD226 has been shown to signal within T cells through Vav1 to enhance TCR-mediated ERK signals and promote IL-17 production (16). By contrast, TIGIT has a cytoplasmic tail with an ITIM and Ig tail-tyrosine-like motif, which reduce TCR expression and signaling (17). Furthermore, TIGIT interferes with CD226 dimerization preventing T cell activation by its presence on the cell surface (5). Thus, TIGIT, when present, plays a dominant role in T cell regulation, while CD226 contributes to immune activation. In addition, TIGIT has been shown to introduce inhibitory signals *via* CD155 on dendritic cells (DCs) and macrophages that render them tolerogenic (4, 18). Thus, expression levels of either TIGIT and/or CD226 on different cell subsets contribute to the ability of CD155 to activate or inhibit cellular functions. TIGIT can be expressed on T cells and NK cells, whereas CD226 can be expressed on T cells, NK cells, B cells, monocytes, and platelets (19–21). T cell subsets express variable levels of TIGIT and CD226. CD226 is highly expressed on recently activated naïve and effector T cells, including Th1, Th2, and Th17 cells, while TIGIT is expressed on exhausted T cells and Tregs (22, 23).

Of note, T follicular helper (Tfh) cells, in particular within germinal centers (GCs), express high levels of TIGIT (22). Tfh cells play a fundamental role in generating humoral immune responses by providing B cell help in the GCs (24, 25). Human Tfh cell differentiation is initiated when CD4^+^ T cells interact with APCs in the presence of cytokines including IL-12, IL-23, and TGF-β, and costimulatory signals *via* OX40 and ICOS, which results in upregulation of the transcription factor Bcl6 (26–30). IL-2 signaling and Blimp-1 are negative regulators of Bcl6 expression and Tfh cell differentiation (31, 32). Pre-Tfh upregulate the expression of ICOS, CXCR5, and PD-1 according to their maturation stage, and accordingly, Tfh cells can be defined by low CD25 expression and high ICOS, CXCR5, and PD-1 (27, 33). Low levels of CD226 and high levels of TIGIT are observed on GC-Tfh cells (22, 34–36). TIGIT expression on circulating Tfh cells in human blood has been associated with high ICOS and IL-21 (37). However, how TIGIT and CD226 regulate the differentiation, maturation, and stability of Tfh cells in humans remains unclear.

In this study, we aimed to determine the biological importance of the CD155/TIGIT/CD226 axis in CD4^+^ T cell differentiation into Tfh cells and for the maintenance of mature GC-Tfh cells. To simplify the stimulatory signals, we constructed L cells constitutively co-expressing CD32 and CD155, which can act as an artificial APC when anti-CD3 is added to the culture. Here we show that CD226 signals promote Tfh cell differentiation on naïve and pre-Tfh human tonsillar cells, however, mature GC-Tfh cells become less dependent on CD226 signals for their activation and maintenance.

## Results

### TIGIT Expression Correlates With a Stable Tfh Signature

**Figure 1:**
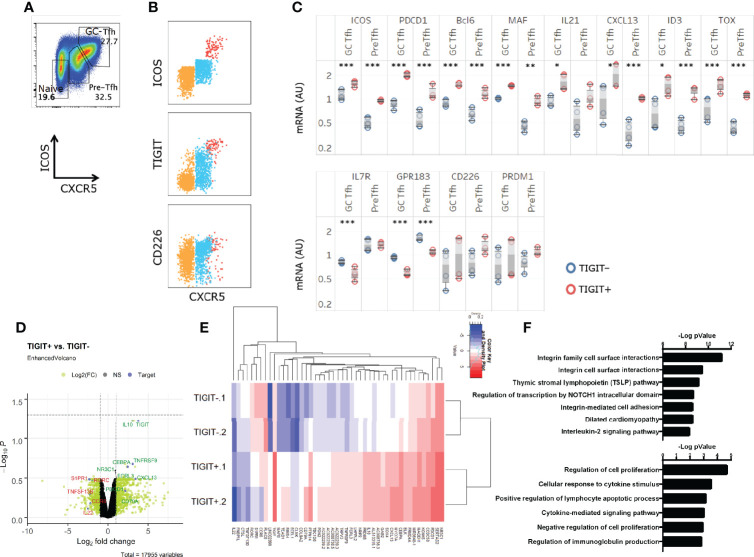
TIGIT^+^GC-Tfh cells express a strong Tfh gene signature. **(A)** Definition of GC-Tfh cells (CXCR5^hi^ICOS^hi^), pre-Tfh cells (CXCR5^lo^ICOS^lo^), and naïve (CXCR5^-^ICOS^-^) in human tonsils in this study. **(B)** TIGIT, CD226, ICOS and CXCR5 expression on tonsillar CD4+ T cell with colors depicting each pre-gated subset (Naïve – Orange, Pre-Tfh – Blue, GC-Tfh – Red). **(C)** GC-Tfh and Pre-Tfh subsets were each sorted according to TIGIT expression, and Tfh-associated gene expression was determined. Paired t-test. *p < 0.05; **p < 0.01; ***p < 0.001. **(D, E)** The transcriptome of TIGIT^+^ and TIGIT^-^ pre-Tfh cells was analyzed by RNAseq. Differentially expressed genes are shown in a volcano plot with selected genes of interest highlighted in green if upregulated and downregulated genes listed in red **(D)** and in a heat map the 47 most significant genes are shown in **(E)**. **(F)** The top differential pathways between TIGIT^+^ and TIGIT^-^ pre-Tfh cells. (top) EnrichR (39) using pathway databases from BioPlanet 2019. (bottom) Biological Process 2018. Data is depicting 2 biological replicates per condition.

Naïve CD4^+^ T cells [CXCR5^-^ICOS^-^ cell population enriched with naïve cells (38)], Tfh precursors (pre-Tfh), or GC-Tfh cells from the human tonsil were identified by the expression level of CXCR5 and ICOS as previously described (38) (**Figure 1A**). In these subsets expression of TIGIT was upregulated on CXCR5^hi^ ICOS^hi^ GC-Tfh cells, as described for murine Tfh cells (36) (**Figure 1B**). An intermediate level of TIGIT expression was exhibited by pre-Tfh cells and low TIGIT expression by CXCR5^-^ICOS^-^ cells (**Figure 1B**). CD226 was co-expressed by approximately half of TIGIT^+^ GC-Tfh cells. A majority of pre-Tfh cells and a fraction of CXCR5-ICOS- cells expressed CD226.

To determine whether TIGIT expression is associated with the maturation stage of human Tfh cells, GC-Tfh cells and pre-Tfh cells were sorted into TIGIT^+^ and TIGIT^-^ subsets and analyzed for the expression of Tfh-related genes by Quantigene. We found that TIGIT^+^ GC-Tfh cells displayed increased *ICOS*, *PDCD1*, and *IL21* and reduced *IL7R*, *KLF2*, and *GPR183* than TIGIT^-^ GC-Tfh cells (**Figure 1C**). Similarly, TIGIT+ pre-Tfh cells expressed stronger Tfh-gene signature than TIGIT- pre-Tfh cells (**Figure 1C**). Further RNA sequencing analysis on sorted cells indicated the association of TIGIT^+^ cells with Tfh genes, such as *PDCD1* and *CXCL13*, and downregulation of *S1PR1, RORC, and CCR6 which* reaffirmed the more mature status of TIGIT^+^ cells (**Figures 1D, E**). Interestingly, *IL10* was found to be substantially higher in TIGIT^+^ cells than TIGIT^-^ cells, which is an inhibitory cytokine associated with suppressive functions of a human Tfh cell subset (40).

The major pathway that differed between TIGIT positive and negative cells was IL-2 signaling pathway (**Figure 1F**). Further analysis showed that the majority of the differentially regulated genes in the IL-2 signaling pathway (such as *S1PR1*, *FLT3LG*, and *MYC*) were downregulated in TIGIT^+^ cells, and described an inhibitory pathway for Tfh development (31), (**Supplemental Table 1**). TIGIT^+^ cells also expressed a gene signature associated with the regulation of cell proliferation (**Figure 1F**). These observations suggest that TIGIT expression correlates with the maturation stage of human Tfh cells and is associated with reduced IL-2 signaling.

### TIGIT Identifies Less Proliferative Tfh Cells

**Figure 2:**
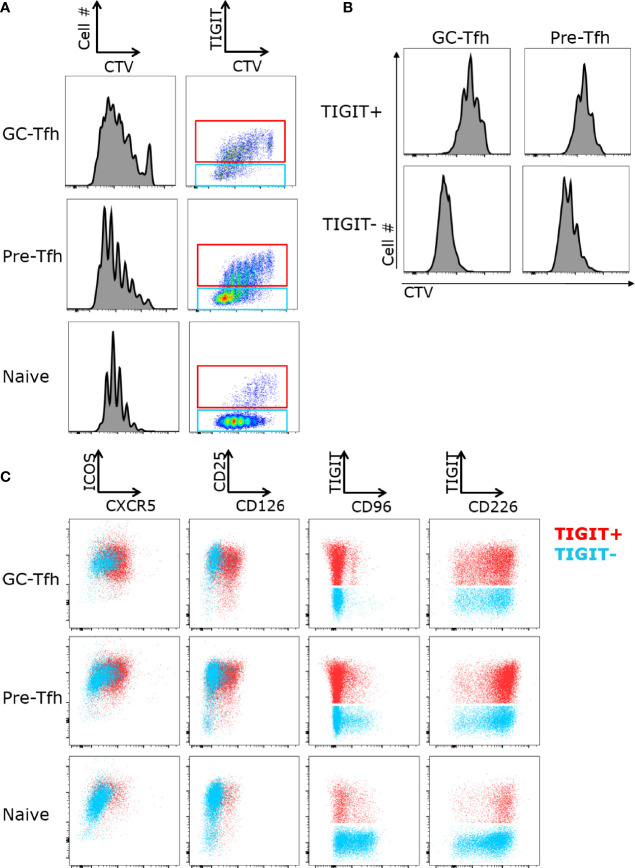
TIGIT^+^ Tfh cells proliferate less upon stimulation. **(A)** FACS-sorted GC-Tfh, pre-Tfh, and naïve cells from tonsils were labeled with Cell Trace Violet (CTV) and stimulated with anti-CD3 and anti-CD28 for 5 days. Cell proliferation and TIGIT expression was analyzed by flow cytometry. **(B)** Proliferation of TIGIT+ and TIGIT- subsets of GC-Tfh and pre-Tfh cells cultured with SEB-pulsed B cells for 5 days. **(C)** Expression of ICOS, CXCR5, CD226, TIGIT, CD96, CD25, and CD126 on sorted GC-Tfh, pre-Tfh and naïve T cells stimulated with anti-CD3 and anti-CD28 for 3 days. Gated to TIGIT^+^ (shown in red) and TIGIT^-^ (shown in blue). Representative FACS plots shown for 3 experiments.

Next, we examined proliferation of tonsil CD4 T cell subsets and whether TIGIT^+^ and TIGIT^-^ cells differently respond to stimulation. Sorted GC-Tfh, pre-Tfh and naive cells were stimulated with anti-CD3 and anti-CD28, and the proliferation of these cells was measured by dilution of Cell Trace Violet with flow cytometry. In all three cell subsets, TIGIT expression was higher on cells that proliferated less (**Figure 2A**). To determine if cells lost TIGIT expression upon proliferation or were less proliferate cells, we sorted TIGIT^+^ and TIGIT- GC-Tfh and pre-Tfh cells, and stimulated with Staphylococcal enterotoxin B (SEB)-pulsed B cells. In both cell subsets, TIGIT^+^ cells proliferated less than TIGIT^-^ cells (**Figure 2B**). This observation corresponds with the gene signature of regulation of cell proliferation in TIGIT^+^ cells (**Figure 1F**). Thus, TIGIT expression is associated with reduced proliferation in Tfh cells.

### Activated GC T Cells Upregulate CD226, but Not CD96

We next determined the expression levels of the CD226/TIGIT family receptors after T cell activation. Naive, pre-Tfh, and GC-Tfh cells were sorted and then activated with anti-CD3 and anti-CD28. While CD226 was expressed at a low frequency on ex vivo tonsillar T cell subsets, all subsets highly expressed CD226 upon activation (**Figure 2C**). CD96 was also highly upregulated on naïve cells and exhibited intermediate expression on pre-Tfh cells with almost no expression on GC-Tfh cells. Additionally, CD96 was not co-expressed with TIGIT, while the majority of TIGIT expressing cells also co-expressed CD226. We also found that TIGIT^+^ GC-Tfh and pre-Tfh cells expressed higher levels of Tfh-associated markers CXCR5 and CD126 (**Figure 2C**). Collectively, while CD226 expression was strongly induced in all subsets, TIGIT expression remained correlated with Tfh markers even after activation.

### Tfh Polarization Does Not Induce TIGIT Expression

**Figure 3:**
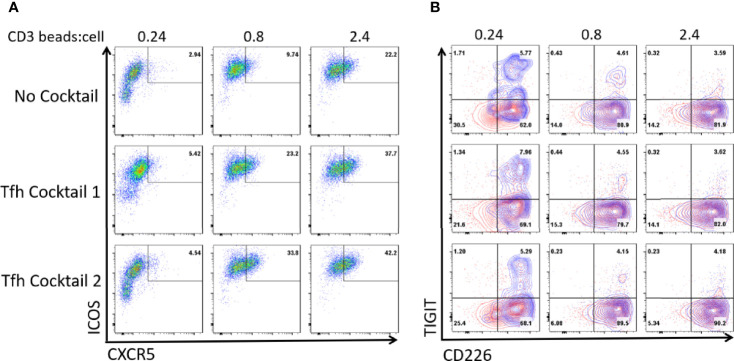
The culture of CD4+ T cells in Tfh-promoting conditions does not induce TIGIT. Total blood CD4+ T cells were cultured in conditions promoting Tfh cell differentiation for 3 days; using Activin A and IL-12 (Tfh cocktail 1) or IL-12, IL-23, rOX40L, and TGF-β (Tfh cocktail 2) in the presence of anti-CD3-CD28 beads at the indicated ratio. **(A)** ICOS and CXCR5 expression of the cultured T cells. **(B)** CD226 and TIGIT expression of the total cultured T cells (outlined in red) and on CXCR5^+^ICOS^+^ cells (overlayed in blue). Representative FACS plots out of 3 experiments.

We and others previously showed that the combination of IL-12 and Activin A or the combination of IL-12, IL-23, OX40L, and TGF-β induce human naïve T cells to become functional Tfh-like cells *in vitro* (27, 28, 30, 41). We examined whether TIGIT is expressed on human blood total CD4^+^ T cells cultured with these Tfh-promoting conditions. We confirmed that these culture conditions promoted the expression of ICOS and CXCR5 in association with an increase of anti-CD3 and anti-CD28-coated beads (**Figure 3A**). TIGIT was expressed on CXCR5^+^ ICOS^+^ cells cultured with few CD3-CD28 beads (**Figure 3B**, indicated by blue contour), however, upon stronger stimulation with increased CD3-CD28 beads, TIGIT expression was diminished. Thus, Tfh-promoting culture conditions did not induce TIGIT, and induction of TIGIT expression on GC-Tfh cells requires additional signals present in the GC environment *in vivo*.

### CD155 and CD122 Are Expressed on Tonsil Monocytes and cDCs But Not B Cells

**Figure 4:**
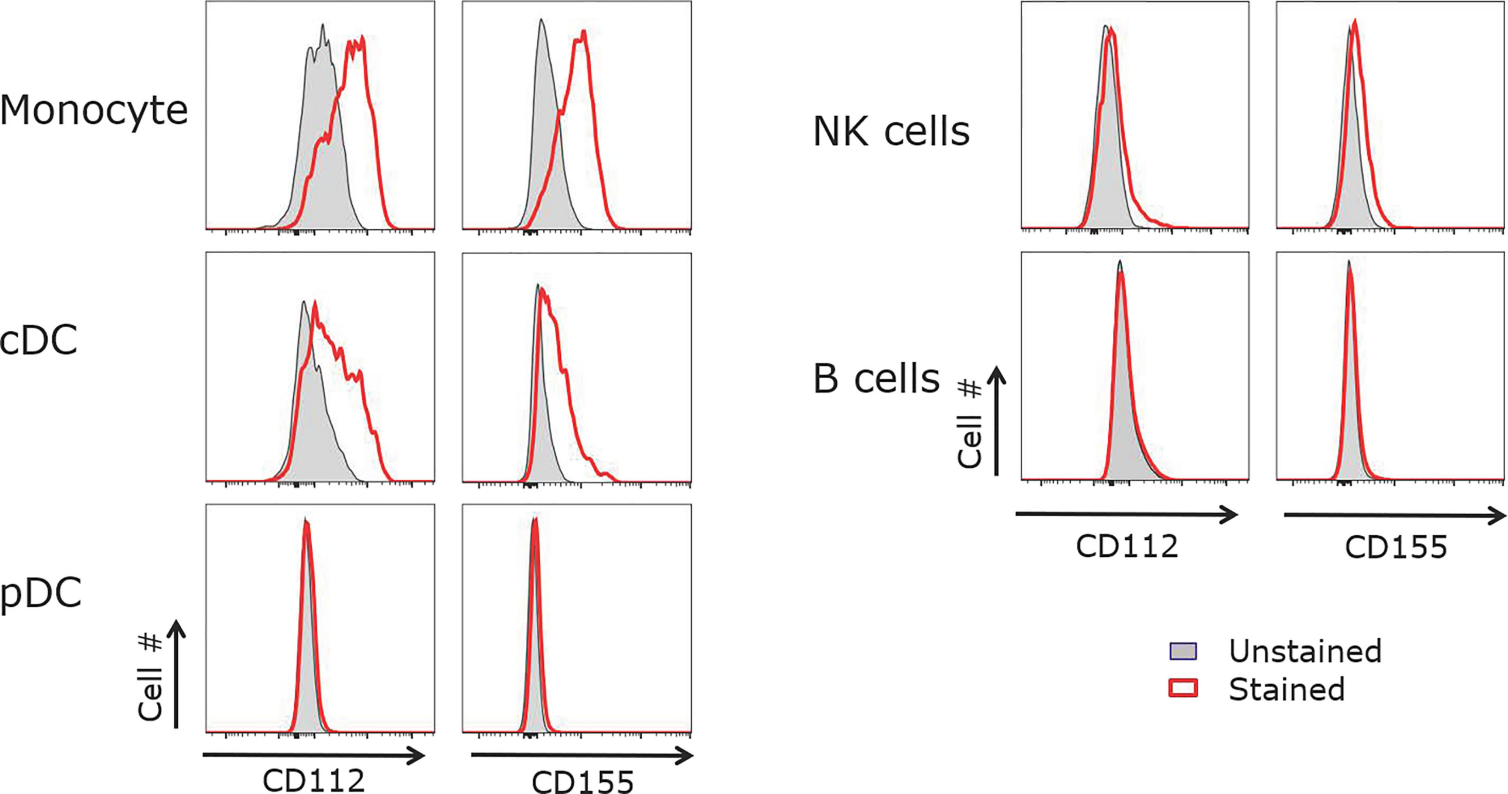
CD155 and CD112 are expressed by monocytes and cDCs but not B cells. Tonsillar APCs were analyzed for CD155 and CD112 expression. Monocytes were defined as CD3^-^CD19^-^CD14^+^, cDCs as CD3^-^CD19^-^MHCII^+^CD11c^+^, pDCs as CD3^-^CD19^-^MHCII^+^CD123^+^, NK cells as CD3^-^CD19^-^, and B cells as CD3^-^CD56^-^CD19^+^. Representative data out of 5 experiments.

To determine the cells expressing the ligands for CD226 and TIGIT in the microenvironment of human tonsils, we examined CD112 and CD155 expression on B cells, monocytes, and DCs. CD155 and the lower affinity receptor CD112 were both expressed on CD14^+^ monocytes and CD11c^+^ cDCs but not pDCs, B cells or NK cells (**Figure 4**). Previous studies have shown that follicular DCs (FDCs) express CD155 (34–36). The lack of CD155 and CD112 expression on B cells suggests that GC-Tfh cells do not receive signals *via* TIGIT and/or CD226 when they interact with B cells near and within GCs. GC response in secondary lymphoid organs is highly dependent on stromal cell network, and GC-Tfh cells and B cells migrate along FDC network in GCs (42). Therefore, within the GC environment, GC-Tfh cells likely receive signals *via* CD226 or TIGIT chronically from FDCs.

### CD155 Costimulation Promotes Tfh Differentiation

**Figure 5:**
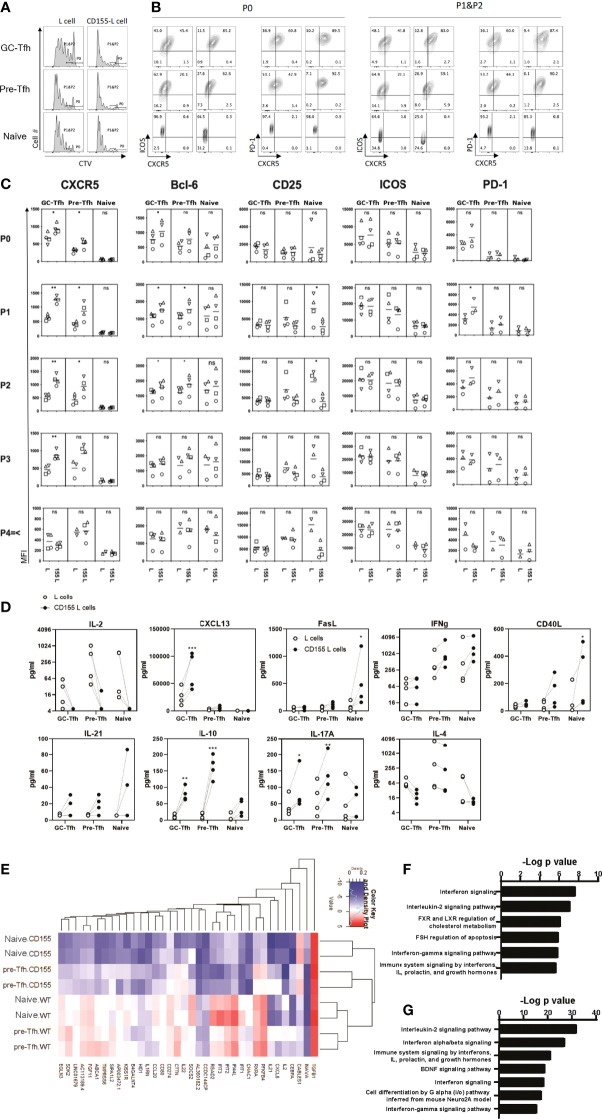
CD155 costimulation promotes the early phase of Tfh cell differentiation. Tonsilar GC-Tfh, pre-Tfh, and naïve cells were stimulated with L cells or CD155 L cells coated with anti-CD3. **(A)** Cell proliferation assessed at day 5 by CTV dilution. **(B)** Phenotype assessed at day 5 by flow cytometry. Per stain each left panel was cultured with L cells; Right panels were cultured with CD155^+^ L cells as denoted in **(A)**. P0: undivided cells, P1&P2: divided cells one and two times. **(C)** CXCR5, Bcl-6, CD25, ICOS, and PD-1 expression was measured in proliferated (P1, P2, P3, and P4≤) and undivided (P0) GC-Tfh, pre-Tfh, and naïve cells stimulated with L cells or CD155^+^ L cells. **(D)** Cytokine concentration in culture supernatants is shown with dotted lines indicating matching donor samples (n= 4, 4 separate experiments). Paired t-test. *p <0.05; **p < 0.01; ***p < 0.001. **(E)** Top 38 most significantly different genes shown between CD155^+^ L cell and L cell stimulated naïve and pre-Tfh cells by RNAseq analysis (n= 2 donors). Pathway analysis for genes from **(F)** pre-Tfh or **(G)** naïve cells that were found to be significantly different when stimulated with L cells or CD155^+^ L cells by RNAseq (enrichR (39): BioPlanet 2019). ns, not significant.

To determine the role of CD155 stimulation on tonsillar CD4^+^ T cells, we generated CD155 expressing L cells. Parental mouse fibroblast L cells express human CD32, which can mount anti-CD3 upon loading. L cells were successfully transfected with a CD155 expressing lentiviral vector (**Supplemental Figure 1**). The culture of GC-Tfh cells with CD155^+^ L cells without anti-CD3 did not largely affect the survival or cell activity as compared to the culture with the parental L cells (data now shown). Therefore, anti-CD3 along with L cells or CD155^+^ L cells were used to activate tonsillar GC-Tfh, pre-Tfh, and naïve CD4^+^ T cells. CD155^+^ L cells induced a robust increase in proliferation in all three cell subsets as compared to parent L cells (**Figure 5A**). Of note, GC-Tfh cells cultured with CD155^+^ L cells lost the expression of TIGIT and CD226 (**Supplemental Figure 2**), probably due to the internalization of these molecules upon interaction with CD155. Thus, CD155 can act as a costimulatory molecule even on GC-Tfh cells and promote cell proliferation. The addition of anti-CD28 did not significantly increase the proliferation of T cells stimulated with CD155^+^ L cells (not shown).

CD155 stimulation induced upregulation of CXCR5 and PD-1 on proliferating GC-Tfh cells (**Figures 5B, C**). Pre-Tfh cells also upregulated CXCR5. In naïve cells, CD155 stimulation reduced CD25 expression. Interestingly, we also observed a strong reduction in the production of IL-2 (**Figure 5D**), the cytokine that inhibits Tfh cell differentiation by activating STAT5 (33). Thus, CD155 stimulation upregulates Tfh markers CXCR5 and Bcl-6 while downregulating IL-2 and CD25 that limit Tfh cell differentiation.

Activated and dividing CD4^+^ T cells change the phenotype according to the number of cell divisions. To assess the effect of CD155 stimulation on the phenotype of CD4^+^ T cells in the early phase of activation, we analyzed the expression of Tfh markers in cells divided less than three times (**Figure 5C**). We found that CD155 stimulation upregulated the expression of CXCR5, Bcl6, and PD-1 on GC-Tfh cells even at undivided and the first-division phases. CD155 stimulation also upregulated CXCR5 and Bcl6 expression on pre-Tfh cells. Notably, CD155 stimulation almost abolished the expression of CD25 at an early activation phase of pre-Tfh cells and naïve T cells.

To determine the functionality of the CD155 stimulated cells, we examined the induction of Tfh associated cytokines and chemokines. In GC-Tfh cells, CD155 stimulation promoted IL-10 and CXCL13 production (**Figure 5D**), both of which are associated with Tfh-driven B cell responses (43, 44). CD155 stimulation on pre-Tfh cells promoted IL-10, IL-17A, and CD40L production. Additionally, CD155 promoted naïve cells to produce FasL and CD40L, which promote B cell survival and antibody production (45). Although not statistically significant, CD155 stimulation tended to increase IL-21 expression by all the subsets. Importantly, CD155 induced all three tonsillar CD4^+^ T cells subsets to inhibit IL-2 production (**Figure 5D**). These data suggest that CD155 stimulation promotes an environment to aid B cell antibody production.

Further investigation into the gene transcription changes induced by CD155 signaling was performed with RNAseq analysis. CD155^+^ L cell treatment induced a greater reduction in gene expression than enhancement compared to L cells (**Figure 5E**). Further confirmation of the role of CD155 treatment displayed in IL-2 signaling was observed by the reduction in *IL2* gene expression and the significance of the IL-2 signaling pathway highlighted from the differentially expressed genes for both pre-Tfh and naïve cells (**Figures 5E**–**G**). In addition, many genes of the interferon signaling pathways were reduced by CD155 treatment resulting in both type I and type II interferon pathways being expressed within the top 7 differentially expressed pathways in both pre-Tfh and naïve cells with CD155 treatment (**Figures 5F, G** and **Supplemental Table 1**). Of note, upregulation of *IL10* gene expression by CD155 stimulation was confirmed. These results indicate that CD155 signaling in tonsillar CD4^+^ T cells can modify cytokine expression patterns.

### CD155 Mediated Costimulation Through the CD226 Pathway

**Figure 6:**
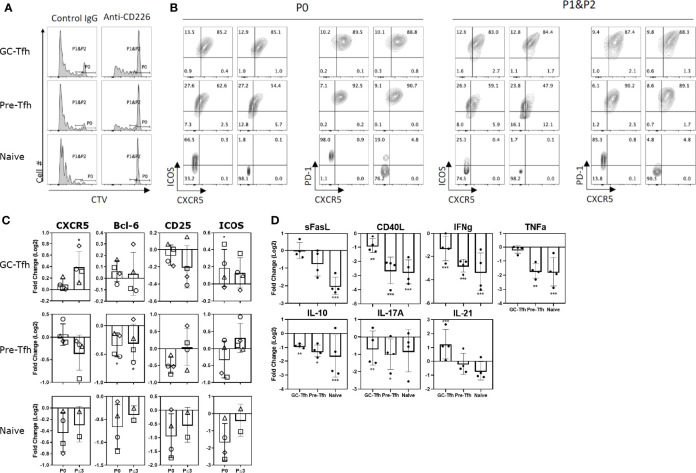
CD155 signaling on T cells is CD226 dependent. Tonsilar GC-Tfh, pre-Tfh, and naïve cells were stimulated with CD155^+^ L cells coated with anti-CD3 in the presence of control IgG or anti-CD226 blocking mAb. **(A)** Cell proliferation assessed at day 5 by CTV dilution. Left panels – cultured with control IgG; Right panels – cultured with anti-CD226. **(B)** Phenotype assessed at day 5 by flow cytometry. Per stain each left panel was cultured with control IgG; Right panels were cultured with anti-CD226 as denoted in **(A)**. P0: undivided cells, P1&P2: divided cells one and two times. **(C)** The fold change in CXCR5, Bcl-6, CD25, and ICOS expression by anti-CD226 treatment compared to control IgG is shown in undivided (P0) and proliferated (P ≤ 3) GC-Tfh, pre-Tfh, and naïve cells. Paired t-test. l*p < 0.05; **p < 0.01. **(D)** The fold change in cytokine concentration by anti-CD226 treatment compared to control IgG is shown. Paired t-test. *p < 0.05; **p < 0.01; ***p < 0.001.

To determine the contribution of CD226 in CD155-mediated costimulation, a CD226 blocking antibody was utilized. Following CD155^+^ L cell and anti-CD3 stimulation, CD226 blockade abrogated the proliferation of naïve T cells and blocked most pre-Tfh proliferation but inhibited GC-Tfh proliferation only modestly (**Figure 6A**). Upon examination of the expression of Tfh markers, with anti-CD226 blocking antibody naïve and pre-Tfh cells showed a reduction in CXCR5, Bcl-6, CD25, and ICOS (**Figures 6B, C**). These observations show that the CD155-CD226 axis promotes the proliferation and the expression of Tfh markers on naïve and pre-Tfh cells. Interestingly, the opposite pattern was seen in GC-Tfh cells. Following CD155 stimulation, the anti-CD226 treated group exhibits a significant increase in CXCR5 and ICOS expression (**Figures 6B, C**). Thus, GC-Tfh cells, which express high levels of TIGIT, are less dependent on the signals *via* CD226 for cell proliferation and the maintenance of Tfh molecules.

We then examined the change in cytokine production by CD226 blockade. CD226 blocking antibody substantially reduced the cytokine production by all three CD4^+^ T cell subsets compared to the control, with the exception of IL-21 by GC-Tfh cells (**Figure 6D**). Unexpectedly, IL-10 production was also reduced by CD226 blocking. Thus, CD226 has a dominant role in the expression of a wide range of cytokines by all the tonsillar CD4^+^ T cell subsets. Increased IL-21 production from GC-Tfh cells by CD226 blocking further supports the notion that GC-Tfh cells are less dependent on CD226 signals.

## Discussion

The relatively increased level of TIGIT expression on tonsillar GC-Tfh cells compared to other T effector cell subsets (22) led us to determine the association between TIGIT expression and the maturation stage of Tfh cells. As compared to TIGIT^-^ compartment, TIGIT^+^ pre-Tfh cells upregulated gene and protein expression of Tfh markers, including IL-21, IL-10, CXCR5, and PD-1, an observation consistent with blood circulating TIGIT^+^ Tfh cells (37). TIGIT^+^ pre-Tfh and naïve cells also exhibited increased expression of CD126 (IL-6R) and reduced genes associated with the IL-2 signaling pathway compared to TIGIT^-^ pre-Tfh cells. Given that IL-6 promotes while IL-2 inhibits Tfh differentiation (31, 46), TIGIT^+^ pre-Tfh cells appear more prone to mature in Tfh cell differentiation. Furthermore, TIGIT expression on tonsillar CD4^+^ T cells is associated with reduced proliferation and increased Tfh gene signature. Thus, our study shows that TIGIT expression is associated with the maturation status of human Tfh cells. Of note, TIGIT expression was not upregulated upon the culture of naïve CD4^+^ T cells in the conditions promoting *in vitro* Tfh differentiation; thus, other pathways must be operative for the upregulation of TIGIT on differentiating Tfh cells *in vivo*.

The lack of CD155 expression on B cells including GC-B cells suggests that TIGIT and CD226 are not directly involved in the interactions between Tfh cells and B cells in GCs. To determine the functional significance of TIGIT and CD226 signals in Tfh cell differentiation, we simplified the culture system by generating L cells constitutively expressing CD155 and CD32 and using these cells as an artificial APC. L cells are derived from murine fibroblasts and thus do not produce human cytokines. Pre-incubation of anti-CD3 with L cells permits its binding to CD32, and CD4^+^ T cells cultured with CD155^+^ L cells receive activation signals only *via* T cell receptor and the TIGIT-CD226 family. Using this system, we found that despite high TIGIT expression on GC-Tfh cells, CD226 plays a dominant role during an early phase of Tfh cell differentiation. Several fundamental differences between naïve and pre-Tfh cells vs. GC-Tfh cells were also noted in the context of CD226 signals.

The culture with CD155^+^ L cells strongly promoted the proliferation of GC-Tfh, pre-Tfh, and naïve CD4^+^ T cells, confirming that CD155 signals act as costimulatory signals in all tonsillar CD4^+^ T cell subsets. We found that CD155 stimulation promoted naïve or pre-Tfh cells to proliferate, produced B helper molecules including IL-10, IL-21, ICOS, and sCD40L, and upregulated Tfh markers including CXCR5 and Bcl6, while inhibited the IL-2-CD25 axis. These changes were largely dependent on CD226 signaling in naïve and pre-Tfh cells. Thus, the CD155-CD226 axis drives the early phase of Tfh cell differentiation. Consistent with our conclusion, murine studies have shown that CD155 or CD226 deficient mice exhibit reduced Tfh cell generation in Peyer’s patches (36, 47).

By contrast, GC-Tfh cells required only weak CD226 signals for cell proliferation. Attenuation of CD226 signals by blocking antibody rather increased the expression of Tfh markers, including CXCR5, ICOS, and Bcl-6 in GC-Tfh cells. Thus, unlike naive and pre-Tfh cells, GC-Tfh cells are less dependent on CD226 signals, and too strong CD226 signals seem detrimental to the integrity of GC-Tfh cells. Herein, high TIGIT on GC-Tfh cells likely contributes to decreasing CD226 signals. Maybe weak and tonic TIGIT signals mediated by interactions with FDCs also contribute to insulating CD226 signals in GC-Tfh cells. Studies in both CD8 T cells and Tregs have noted an association with the ratio of CD226 to TIGIT expression and cell activity (5, 48, 49). While these previous studies focused on the ability of TIGIT blockade to restore CD226 activation, we have noted that this ratio also impacts the ability of CD226 blockade to inhibit T cell activation.

CD226 and TIGIT are primarily considered as an analogy of CD28 and CTLA-4. CD28 and CD226 are expressed on activated naïve and pre-Tfh cells, while CTLA-4 and TIGIT are highly expressed on GC-Tfh cells (22, 50). However, their roles might be somewhat different in the context of Tfh cell differentiation. CD28 plays a role in Tfh cell differentiation until CTLA-4 is upregulated by GC-Tfh cells (51), whereas the importance of CD28 signals for GC-Tfh cells is unclear (52). Similarly, CD226 contributes to Tfh cell differentiation for naïve and pre-Tfh cells until TIGIT is upregulated by GC-Tfh cells. The main difference between CD28 and CD226 signals is that the latter eventually shuts off IL-2 production, whereas CD28 promotes IL-2 production (53). TIGIT signals also inhibit IL-2 production by human CD4^+^ T cells (2). Thus, CD226 and TIGIT seem to contribute to Tfh cell differentiation by inhibiting IL-2 production, at least in humans.

Collectively, our study shows that CD226 and TIGIT collaborate in Tfh cell differentiation and maturation in humans. TIGIT seems important to attenuate CD226 signals on GC-Tfh cells, which express CD226 upon TCR activation. Our study may provide novel insights into the mechanism for controlled human Tfh cell response and the alterations in this axis in the pathogenesis of human autoimmune diseases. Blocking CD226 signals might decrease the generation of Tfh cells and their precursors in patients with autoimmune diseases. However, this needs to be tested with caution, as CD226-blocking might increase the stability of the differentiated mature GC-Tfh cells, contributing to the generation of autoantibodies.

## Materials and Methods

### Cell Culture Reagents

RPMI 1640 media, L-glutamine, Penicillin-Streptomycin, sodium pyruvate, MEM non-essential amino acid solution and HEPES were purchased from Thermo Fisher Scientific (Waltham, MA). Fetal bovine serum, 2-mercaptoethanol, and Mitomycin C (MMC) were purchased from Sigma-Aldrich (Munich, Germany). Staphylococcus aureus Enterotoxin B (SEB) was from Toxin Technology, Inc. (Sarasota, FL). Recombinant human IL-12, IL-23 and TGF-β were purchased from PeproTech (Rocky Hill, NJ). Recombinant human/mouse/rat activin A and soluble OX40L (sOX40L) were purchased from R&D Systems, Inc. (Minneapolis, MN). Cell lines and PBMCs were cultured in complete RPMI medium (RPMI-1640 medium containing 2 mM L-glutamine, 100 U/ml penicillin, 100 μg/ml streptomycin, 1 mM sodium pyruvate, 1X MEM non-essential amino acid, 25mM HEPES and 50 μM 2-mercaptoethanol supplemented with 10% fetal bovine serum) in the presence of 5% CO_2_ in a humidified atmosphere at 37°C.

### Generation of CD155-L Cells

Mouse fibroblastic L cells stably transfected with the human CD32 (CD32-L cells) was kindly provided by Dr. Yong-Jun Liu (54). For generation of L cells stably expressed human CD32 and human CD155 (CD32/CD155-L cells), human CD155 gene was transduced into CD32-L cells by using lentiviral vector. A human CD155 alpha CDS 300-1553 expressing pLenti6.3/V5-DEST was purchased from Thermo Fisher Scientific. The vector was transfected to HEK293T cells using Pantropic Lentiviral Packaging System (Cell Biolabs, Inc., San Diego, CA) and PEI max (Polysciences, Inc., Warrington, PA). The supernatants were collected after 72 hours. CD32-L cells were infected with the supernatants by spin infection for 2 hours. After 72 hours, CD155 and CD32 double expressing L cells were selected by sorting and Mitomycin C selection.

### Flow Cytometry

For immunophenotyping, cells were stained with LIVE/Dead dye and Fc blocked prior to staining. Then, cells were stained with fluorochrome-conjugated antibodies in FACS buffer (PBS with 2% heat inactivated FBS) or Brilliant Stain Buffer (BD). Staining of transcription factor Bcl-6 was performed with Tru-Nuclear Transcription Factor Buffer Set (BioLegend). Stained cells were acquired on LSRII (BD Biosciences, NJ) or Cytek Aurora (Cytek Biosciences Inc. CA)) and the flow data were analyzed with FlowJo (BD). The antibodies and viability dyes used are listed in the **Supplemental Table 2**.

### Isolation of CD4^+^ T Cell and B Cell Subsets From PBMC and Tonsillar Cells

To purify naïve (CD4^+^CD45RO^-^) and memory (CD4^+^CD45RO^+^) blood CD4^+^ T cells, thawed PBMCs were magnetically isolated by human CD4^+^ T cell isolation kit and human CD45RO Microbeads (Miltenyi Biotech, Bergisch Gladbach, Germany) according to the manufacturer’s protocol.

To obtain B cells, B cell subsets and CD4^+^ T cell subsets from tonsillar cells, thawed cells were separated into CD19^+^ and CD19^-^ fractions by EasySep Human CD19 Positive Selection Kit II (Stemcell Technologies) using double-triple dose of antibody and beads in manufacturer’s protocol. To purify B cells, CD19^+^ fraction was stained with fluorescence conjugated antibodies and viability dye and Live/Dead^-^CD20^+^CD3^-^ CD14^-^CD56^-^ cells with FACSAria. To isolate CD4^+^ T cell subsets, CD19^-^ fraction (T cell-enriched fraction) was stained with fluorescence conjugated antibodies and viability dye and sorted into CXCR5^hi^ICOS^hi^ (GC-Tfh), CXCR5^lo^ICOS^lo^ (pre-Tfh) and CXCR5^-^ICOS^-^ (naïve) subsets from Live/Dead^-^CD4^+^CD8^-^CD20^-^CD11c^-^CD14^-^ CD56^-^CD123^-^ cells with FACSAria. In some experiments, T cell-enriched fraction was sorted into smaller populations according to TIGIT expression.

If required, sorted populations were labeled with 2.5μM CTV (Cell Trace Violet, Thermo Fisher Scientific) according to manufacturer’s instruction.

### *In Vitro* Tfh Polarization of Blood CD4^+^ T Cells

Blood naïve (CD45RO^-^) CD4^+^ T cells (5 x10^4^/well) were cultured in 96-well round bottom and activated by Dynabeads Human T-Activator CD3/CD28 (Thermo Fisher Scientific) at various bead: cell ratios. After overnight, a combination of activin A (50 ng/ml) and IL-12 (100 pg/ml) (Tfh Cocktail 1); or a combination of IL-12 (100 pg/ml), sOX40L (100 ng/ml), IL-23 (25 ng/ml), and TGF-β (5 ng/ml) (Tfh Cocktail 2) was added. After three days culture, the cells were analyzed for assessing the expression of surface molecules by flow cytometry.

### *In Vitro* Tonsillar Cell Stimulation With CD3/CD28 Dynabeads

Tonsillar GC-Tfh, pre-Tfh or naïve cells (5 x10^4^/well) were cultured in 96-well round bottom plates in the presence of Dynabeads Human T-Activator CD3/CD28 at a bead: cell ratio 0.16 for 3-5 days. Phenotype were analyzed by flow cytometry.

### Tonsillar T Cell–B Cell Coculture Assay

Sorted TIGIT+ or TIGIT- GC-Tfh or pre-Tfh cells were co-cultured with tonsillar B cells in 96-well round bottom plates (2 x 10^4^ cells/well) in the presence of 0.1 ug/ml of SEB. In the presence of 0.1 μg/ml of SEB. Cells were cultured for 5 days and analyzed by flow cytometry.

### *In Vitro* Tonsillar T Cell Stimulation With L Cells

On day -1, L cells and CD155-L cells were treated 4 μg/ml MMC for 3 hours. Cells were collected by Trypsin-EDTA (Thermo Fisher Scientific), washed 2 times and transferred to 96-well flat bottom plates (2.5 x 10^4^/well). On day 0, anti-human CD3 (OKT3; prepared ‘in house’) were added at final concentration of 0.2 - 0.5 μg/ml and plates were incubated at 37°C for at least an hour. Sorted (and CTV-labeled if indicated) GC-Tfh, pre-Tfh and naïve cells were added to the plate (5.0 x 10^4^/well) and cultured for 3-5 days for further analysis.

In some experiments, control IgG (30 μg/ml), or blocking anti-CD226 (10 μg/ml; LeoA1; Millipore Sigma) antibodies were added on day 0 (and day 2 and day 4).

### Cytokine Array

CD4^+^ T cell subsets were co-cultured with L or CD155-L cells. On day 5, supernatant was collected for determination of cytokine levels. The following cytokines were quantified by ProcartaPlex Immunoassay BLC, CD40L, FAS-L, IFNg, IL-1b, IL-2, IL-4, IL-6, IL-9, IL-10, IL-17A, IL-21, IL-27, IL-31, TNFa (Thermo Fisher) according to the manufacturers’ instructions. Data was collected using the Luminex 200

### QuantiGene Plex Assay

TIGIT^+^ and TIGIT^-^ GC-Tfh cells were sorted by FACSAria. The sorted cells were directly collected into the lysis mixture and prepared the cell lysates by using QuantiGene Sample Processing Kit (Thermo Fisher Scientific). The expression levels of mRNA were analyzed with using QuantiGene Plex Assay kit (Thermo Fisher Scientific) and MAGPIX system (Luminex Corporation, Austin, Texas) according to the manufactures’ protocols. Fold changes to average of three house-keeping genes were calculated after subtraction of background.

### RNAseq Analysis

Sorted and CTV-labeled GC-Tfh, pre-Tfh and naïve were co-cultured with L cells or CD155-L cells. On day3, cells were collected, stained with APC-Cy7-CD4^+^ and PI and sorted live CD4^+^CTV^+^ cells by FACSAria. Total RNA was extracted from sorted cells by Qiagen RNAeasy kit and RNA sequencing was run by Genewiz Inc.

Raw reads were aligned with STARaligner v2.7.0f against the human genome GRCh38.v27 with outFilterScoreMinOverLread = 0.3, outFilterMatchNminOverLread = 0.3, alignSJoverhangMin = 8, alignSJDBoverhangMin = 1. Otherwise default parameters were used. Reads were summarized and assigned to genomic features by feature Counts v2.0.1. Raw counts were further processed using the edgeR v3.30.0/limma v3.4.4.1 pipeline with R v4.0.0 to normalize counts, for quality control, and to identify differentially expressed genes. Genes were considered significantly differentially expressed with a fold change (FC) of |FC| ≥ 2 and a P-value ≤ 0.05 (adjusted for multiple testing after Benjamini & Hochberg). Genes and groups in heatmaps were clustered using their expression values (deviating from the collective median expression) together with “single linkage method” (which is closely related to the minimum spanning tree).

Significantly differentially expressed genes (see *Statistical Analysis*) from each comparison was entered into enrichR (39) and the top pathways from BioPlanet2019 and Biological Process 2018 databases are shown sorted by P-value rankings. EnrichR employs a modified Fisher’s Exact Test to calculate a rank score (Z-score) of enriched gene sets. The p-values are adjusted for multiple testing after Benjamini & Hochberg.

### Statistical Analysis

The significance of the difference between groups in the experiments was evaluated by two-tailed paired t test and one-way ANOVA with GraphPad Prism (GraphPad Software, San Diego, CA). A value of p < 0.05 was considered significant.

### Study Approval

All human studies described here were approved by the Institutional Review Board of Icahn School of Medicine at Mount Sinai. Buffy coat from healthy donors was obtained from the New York Blood Center, and PBMCs were isolated *via* gradient centrifugation with Ficoll (Stemcell Technologies, Vancouver, British Columbia, Canada). Tonsil samples were obtained from the Cooperative Human Tissue Network and single cells were collected by mechanical disruption. The donors (or their parents or the guardians) have provided an informed consent. PBMCs and tonsillar cells were cryopreserved for further experiments.

## Data Availability Statement

The datasets presented in this study can be found in online repositories. The names of the repository/repositories and accession number(s) can be found below: NCBI GEO, accession no: GSE194293.

## Ethics Statement

All human studies described here were approved by the Institutional Review Board of Icahn School of Medicine at Mount Sinai. Written informed consent to participate in this study was provided by the participants’ legal guardian/next of kin.

## Author Contributions

MY conducted experiments and acquired and analyzed data. AC conducted experiments, acquired and analyzed data, drafted the initial manuscript and revised the manuscript. NI conducted experiments, acquired and analyzed data, drafted manuscript methods and revised the manuscript. CF drafted manuscript methods and analyzed data. NM-O designed research studies and revised the manuscript. GC oversaw the research, assisted with experimental design, and revised the manuscript. HU conceived and oversaw the research, designed research studies, drafted manuscript methods, and revised the manuscript. All authors approved the final manuscript as written.

## Funding

This research was supported by the collaborative fund from EMD Serono. The funder also involved in the study design, collection of RNAseq study data, and the decision to submit the manuscript for publication.

## Conflict of Interest

Authors AC, NM-O and GC were employed by EMD Serono Research and Development Institute Inc. during the project and have equity interest.

The remaining authors declare that the research was conducted in the absence of any commercial or financial relationships that could be construed as a potential conflict of interest.

## Publisher’s Note

All claims expressed in this article are solely those of the authors and do not necessarily represent those of their affiliated organizations, or those of the publisher, the editors and the reviewers. Any product that may be evaluated in this article, or claim that may be made by its manufacturer, is not guaranteed or endorsed by the publisher.
