# Supplementary material for: CD226 and TIGIT Cooperate in the Differentiation and Maturation of Human Tfh Cells
Source: Front Immunol. 2022 Feb 22;13:840457. doi: 10.3389/fimmu.2022.840457 (PMC8902812; doi:10.3389/fimmu.2022.840457)
Supplement: Supplementary file 1 [file DataSheet_1.pdf]

## Supplementary Information

## Supplemental Table 1. RNA sequencing analysis

Significantly different genes from the indicated comparisons that were identified by pathways analysis are listed to observe positive and negative trends. Data was collected by RNAseq and analyzed through enrichR(1): BioPlanet 2019.

| Pre-Tfh (TIGIT+/TIGIT-) | | | |
|---|---|---|---|
| IL-2 signaling | | Regulation of cell Proliferation | |
| Gene | Log Fc | Gene | Log Fc |
| S1PR1 | -2.3359 | FLT3LG | -1.618559071 |
| CASP10 | -2.2310 | MYC | -1.413116242 |
| EPHA4 | -1.9466 | PTPRJ | 1.156554418 |
| RASGRP2 | -1.6558 | EFNB2 | 1.494039059 |
| FLT3LG | -1.6186 | SMAD6 | 1.608984364 |
| ITGB7 | -1.5988 | GAB2 | 1.626926657 |
| FLOT1 | -1.4294 | ZNF703 | 1.73594544 |
| MYC | -1.4131 | TBC1D8 | 1.910953402 |
| LDHB | -0.7445 | BLK | 2.105466742 |
| GAB2 | 1.6269 | CEBPA | 2.446614589 |
| TBC1D8 | 1.9110 | WNK2 | 2.601390655 |
| IL10 | 3.0231 | PTHLH | 2.697879933 |
| | | IL10 | 3.023117032 |
| | | TNFRSF9 | 3.074799046 |
| | | PTPN14 | 4.49690447 |
| Avg | -0.7011 | Avg | 1.7965 |
| Median | -1.5141 | Median | 1.9110 |

| Pre-Tfh (L cell/CD155 L cell) | | | |
|---|---|---|---|
| Interferon signaling | | IL-2 signaling | |
| Gene | Log Fc | Gene | Log Fc |
| XAF1 | -2.9188 | S1PR1 | -2.3359 |
| GBP6 | -2.0910 | CASP10 | -2.2310 |
| MT2A | 1.7960 | EPHA4 | -1.9466 |
| | | RASGRP2 | -1.6558 |
| | | FLT3LG | -1.6186 |
| | | ITGB7 | -1.5989 |
| | | FLOT1 | -1.4294 |
| | | MYC | -1.4131 |
| | | LDHB | -0.7445 |
| | | GAB2 | 1.6269 |
| | | TBC1D8 | 1.9110 |
| | | IL10 | 3.0231 |
| Avg | -1.0713 | Avg | -0.7011 |
| Median | -2.0910 | Median | -1.5141 |

| Naïve Cells (L cell/CD155 L cell) | | | | | |
|---|---|---|---|---|---|
| IL-2 signaling | | | | Interferon alpha/beta signaling | |
| Gene | Log Fc | Gene | Log Fc | Gene | Log Fc |
| MT3 | -4.9540 | NFATC3 | 0.6316 | IFIT3 | -5.0340 |
| IFI44 | -4.7506 | MTHFD1 | 0.6717 | IFIT1 | -4.3509 |
| IL13 | -4.6507 | SAMM50 | 0.6852 | IFIT2 | -4.0812 |
| IFIT1 | -4.3509 | PPP2R5D | 0.6918 | MX1 | -3.2005 |
| SOCS2 | -4.0266 | ARL6IP1 | 0.7133 | IRF7 | -2.9614 |
| PRSS23 | -3.7015 | ACTB | 0.7272 | SOCS3 | -2.7736 |
| HSF4 | -3.3086 | TAX1BP3 | 0.7369 | ISG20 | -2.7261 |
| MX1 | -3.2005 | DOCK8 | 0.7694 | OAS1 | -2.7094 |
| MXI1 | -2.8425 | PIK3CD | 0.8028 | OASL | -2.5749 |
| FOSL2 | -2.7982 | ITPKB | 0.8382 | MX2 | -2.4167 |
| VEGFA | -2.7945 | LAPTM5 | 0.8689 | IFI6 | -2.2422 |
| SOCS3 | -2.7736 | COG4 | 0.9015 | SOCS1 | -2.0717 |
| ANK2 | -2.4425 | STK38 | 0.9104 | IRF8 | -1.8284 |
| LTA | -2.4219 | TTLL1 | 0.9149 | IRF9 | -1.7400 |
| LIF | -2.4117 | PTPN7 | 0.9420 | XAF1 | -1.6981 |
| TNFRSF11A | -2.3108 | LCK | 0.9802 | OAS2 | -1.1389 |
| MYC | -2.2292 | PDE7A | 1.0075 | IFNAR2 | 1.1019 |
| SOCS1 | -2.0717 | RGS10 | 1.0176 | | |
| RGS16 | -1.9586 | SRGN | 1.0281 | | |
| DUSP5 | -1.9553 | CTDSP2 | 1.0334 | | |
| NAMPT | -1.8731 | ESYT1 | 1.0536 | | |
| PMAIP1 | -1.7886 | CTSC | 1.0670 | | |
| IRF9 | -1.7400 | CD27 | 1.1163 | | |
| TERT | -1.7066 | ADA | 1.1375 | | |
| SLC2A3 | -1.7065 | PDE4DIP | 1.1440 | | |
| FRMD4B | -1.5448 | IL16 | 1.2078 | | |
| TNF | -1.5429 | SELL | 1.2181 | | |
| NR4A2 | -1.5178 | INPP5D | 1.2634 | | |
| ASNS | -1.4720 | ITGB7 | 1.2685 | | |
| WARS | -1.4438 | EHD1 | 1.4450 | | |
| ICAM1 | -1.4404 | CD52 | 1.5542 | | |
| NFIX | -1.4282 | SLC25A20 | 1.5542 | | |
| XBP1 | -1.3466 | MT2A | 1.5621 | | |
| CD68 | -1.2900 | SUOX | 1.6082 | | |
| BCL2 | -1.2348 | CD96 | 1.7161 | | |
| UPP1 | -1.2295 | MT1E | 1.7571 | | |
| CYP51A1 | -1.1977 | TCF7 | 1.7893 | | |
| PRKCE | -1.1452 | CD244 | 1.8046 | | |
| DUSP3 | -1.0855 | FCGBP | 1.8789 | | |
| BNIP3L | -1.0457 | CTSW | 2.1736 | | |
| STAT3 | -0.9741 | RASGRP2 | 2.2226 | | |
| SPN | -0.7209 | AQP3 | 2.2642 | | |
| | | MYO1F | 2.7117 | | |
| AVG | | | -0.4593 | Avg | -2.4968 |
| Median | | | 0.6316 | Median | -2.5749 |

**Supplemental Table 2. Flow cytometry reagents used in publication.**

| Antigen / Reagent | Fluorochrome | Clone | Isotype | Company | Catalog # |
|---|---|---|---|---|---|
| CD3 | PerCP-Cy5.5 | UCHT1 | mIgG1 k | BioLegend | 300430 |
| CD3 | PE-Cy7 | SK7 | mIgG1 k | BD | 557851 |
| CD3 | AF700 | UCHT1 | mIgG1 k | BioLegend | 300424 |
| CD3 | BV785 | UCHT1 | mIgG1 k | BioLegend | 300472 |
| CD4 | AF700 | RPA-T4 | mIgG1 k | BioLegend | 300526 |
| CD4 | APC-Cy7 | RPA-T4 | mIgG1 k | BioLegend | 300518 |
| CD4 | APC-Cy7 | OKT4 | mIgG2b k | BioLegend | 317418 |
| CD8 | AF700 | RPA-T8 | mIgG1 k | BD | 557945 |
| CD8 | APC | SK1 | mIgG1 k | Thermo Fisher | 17-0087-42 |
| CD8 | PacBlue | RPA-T8 | mIgG1 k | BD | 558207 |
| CD11c | PE-Cy7 | B-ly6 | mIgG1 k | BD | 561356 |
| CD14 | APC | M5E2 | mIgG2a k | BD | 555399 |
| CD14 | APC-Cy7 | 63D3 | mIgG1 k | BioLegend | 367107 |
| CD19 | BV750 | HIB19 | mIgG1 k | BioLegend | 302261 |
| CD20 | FITC | 2H7 | mIgG2b k | BioLegend | 302304 |
| CD20 | BV421 | 2H7 | mIgG2b k | BioLegend | 302330 |
| CD25 | APC-Cy7 | M-A251 | mIgG1 k | BioLegend | 356112 |
| CD45RO | APC | UCHL1 | mIgG2a k | BioLegend | 304210 |
| CD56 | APC | B159 | mIgG1 k | BD | 555518 |
| CD56 | BV570 | 5.1H11 | mIgG1 k | BioLegend | 362539 |
| CD96 | PE/Dazzle | NK92.39 | mIgG1 k | BioLegend | 338414 |
| CD112 | PerCP-Cy5.5 | TX31 | mIgG1 k | BioLegend | 337415 |
| CD123 | BV421 | 6H6 | mIgG1 k | BioLegend | 306018 |
| CD126 | BV421 | M5 | mIgG1 k | BD | 566244 |
| CD155 (PVR) | BV605 | SKII.4 | mIgG1 k | BD | 748276 |
| CD185 (CXCR5) | AF488 | RF8B2 | Rat IgG2a k | BD | 558112 |
| CD185 (CXCR5) | AF647 | RF8B2 | Rat IgG2b k | BD | 555813 |
| CD226 (DNAM-1) | APC | 11A8 | mIgG1 k | BioLegend | 338312 |
| CD278 (ICOS) | PerCP-Cy5.5 | C398.4A | Ham IgG | BioLegend | 313517 |
| CD278 (ICOS) | PE-Cy7 | C398.4A | Ham IgG | BioLegend | 313520 |
| CD279 (PD-1) | PE | EH12.2H7 | mIgG1 k | BioLegend | 329906 |
| CD279 (PD-1) | BV421 | EH12.2H7 | mIgG1 k | BioLegend | 329920 |
| TIGIT (VSTM3) | PE | A15153G | mIgG2a k | BioLegend | 372704 |
| MHC II / HLA-DR | AF488 | L243 | mIgG2a k | BioLegend | 307620 |
| Bcl-6 | AF647 | K112-91 | mIgG1 k | BD | 561525 |
| Zombie Red Fixable Viability Kit | | | | BioLegend | 423110 |
| Fixable Viability Dye eFluor™ 780 | | | | Thermo Fisher | 65-0865-14 |
| LIVE/DEAD™ Fixable Aqua Dead Cell Stain Kit | | | | Invitrogen | L34957 |
| Propidium Iodide | | | | Invitrogen | P3566 |
| Human TruStain FcX™ | | | | BioLegend | 422302 |
| CellTrace™ Violet Cell Proliferation Kit | | | | Invitrogen | C34557 |
| Brilliant Stain Buffer | | | | BD | 563794 |
| Tru-Nuclear Transcription Factor Buffer Set | | | | BioLegend | 424401 |

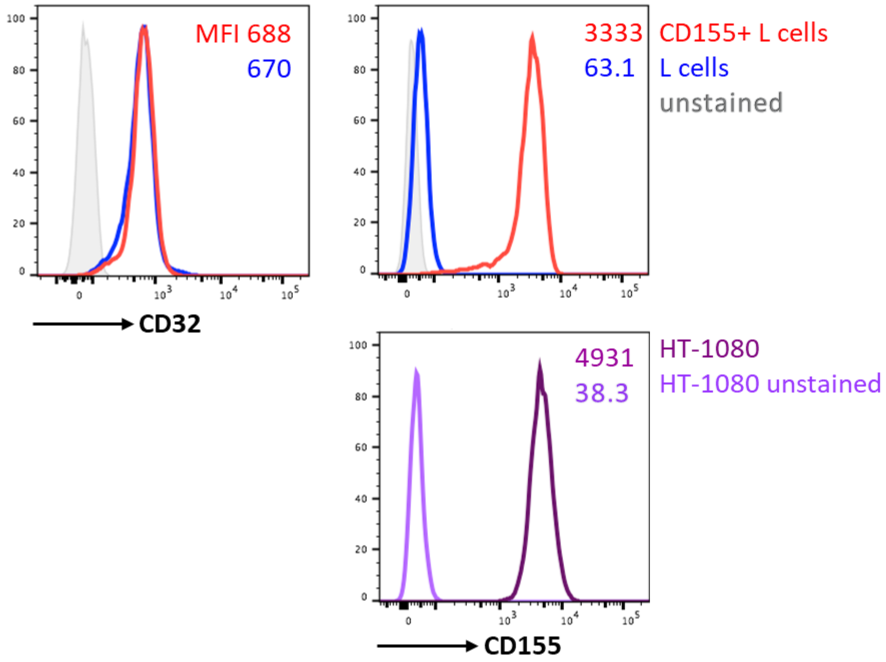


**Supplemental Figure 1. Generation of CD155 expressing L cells.**

CD32+ L cells were transfected with a CD155 expressing lentiviral vector. CD155+ L cells were sorted according to the expression levels of CD155 and CD32. HT-1080 cell line was used as a positive control for CD155 expression.

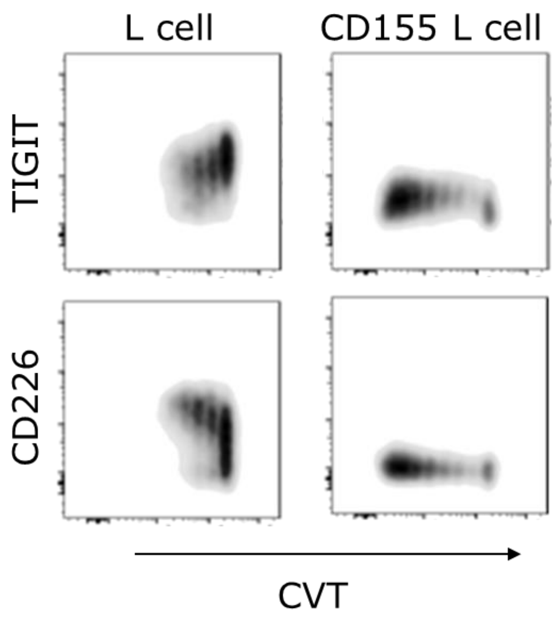


**Supplemental Figure 2. Loss of TIGIT and CD226 expression by culturing with CD155$^{+}$ L cells.**

CVT-labeled GC-Tfh cells were cultured for 5 days either with L cells or CD155$^{+}$ L cells, and the expression of TIGIT and CD226 was analyzed. A representative of 3 experiments.

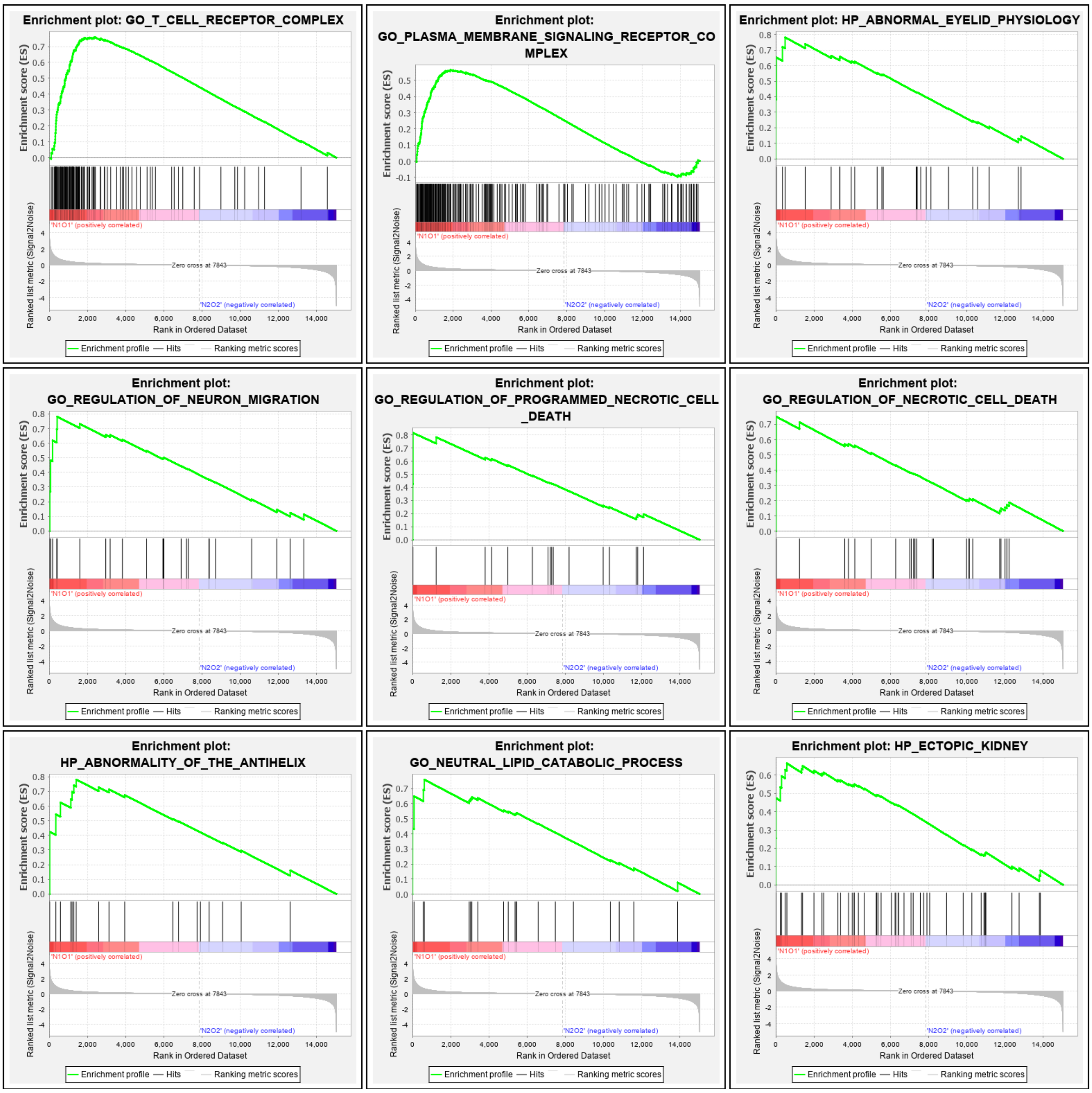


**Supplemental Figure 3. Snapshots of best nine upregulated pathways between TIGIT$^+$ and TIGIT$^-$ pre-Tfh cells after GSEA(2) using the C5 ontology gene sets**

References:


1. Xie Z, Bailey A, Kuleshov MV, Clarke DJB, Evangelista JE, Jenkins SL, et al. Gene Set Knowledge Discovery with Enrichr. Curr Protoc. 2021;1(3):e90.
2. Subramanian A, Tamayo P, Mootha VK, Mukherjee S, Ebert BL, Gillette MA, et al. Gene set enrichment analysis: a knowledge-based approach for interpreting genome-wide expression profiles. Proceedings of the National Academy of Sciences of the United States of America. 2005;102(43):15545-50.
